# *N*′-[1-(4-Chloro­phen­yl)ethyl­idene]acetohydrazide

**DOI:** 10.1107/S1600536810042546

**Published:** 2010-10-23

**Authors:** Huan-mei Guo, Qian Wu, Jie Yang, Yang-chun Liu

**Affiliations:** a Microscale Science Institute, Weifang University, Weifang 261061, People’s Republic of China; b Department of Chemistry and Chemical Engineering, Weifang University, Weifang 261061, People’s Republic of China

## Abstract

In the title compound, C_10_H_11_ClN_2_O, the dihedral angle between the acetohydrazide group and the aromatic ring is 33.76 (9)°. In the crystal, inversion dimers linked by pairs of N—H⋯O hydrogen bonds generate *R*_2_^2^(8) loops.

## Related literature

For a related structure, see: Li & Jian (2008 ▶).
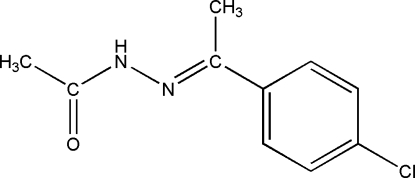


## Experimental

### 

#### Crystal data

C_10_H_11_ClN_2_O
*M* *_r_* = 210.66
Monoclinic, 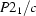

*a* = 15.944 (3) Å
*b* = 5.0061 (10) Å
*c* = 13.950 (3) Å
β = 109.45 (3)°
*V* = 1049.9 (4) Å^3^
*Z* = 4
Mo *K*α radiation
μ = 0.33 mm^−1^
*T* = 293 K
0.25 × 0.20 × 0.20 mm

#### Data collection

Bruker SMART CCD diffractometer
9225 measured reflections
2368 independent reflections
1840 reflections with *I* > 2σ(*I*)
*R*_int_ = 0.028

#### Refinement

*R*[*F* ^2^ > 2σ(*F* ^2^)] = 0.046
*wR*(*F* ^2^) = 0.164
*S* = 1.22
2368 reflections
143 parameters
H atoms treated by a mixture of independent and constrained refinement
Δρ_max_ = 0.29 e Å^−3^
Δρ_min_ = −0.22 e Å^−3^

### 

Data collection: *SMART* (Bruker, 1997 ▶); cell refinement: *SAINT* (Bruker, 1997 ▶); data reduction: *SAINT*; program(s) used to solve structure: *SHELXS97* (Sheldrick, 2008 ▶); program(s) used to refine structure: *SHELXL97* (Sheldrick, 2008 ▶); molecular graphics: *SHELXTL* (Sheldrick, 2008 ▶); software used to prepare material for publication: *SHELXTL*.

## Supplementary Material

Crystal structure: contains datablocks global, I. DOI: 10.1107/S1600536810042546/hb5687sup1.cif

Structure factors: contains datablocks I. DOI: 10.1107/S1600536810042546/hb5687Isup2.hkl

Additional supplementary materials:  crystallographic information; 3D view; checkCIF report

## Figures and Tables

**Table 1:** Hydrogen-bond geometry (Å, °)

*D*—H⋯*A*	*D*—H	H⋯*A*	*D*⋯*A*	*D*—H⋯*A*
N2—H2*A*⋯O1^i^	0.93 (2)	2.02 (2)	2.9384 (18)	170.3 (18)

Symmetry code: (i) 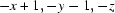.

**Fig. 1.**
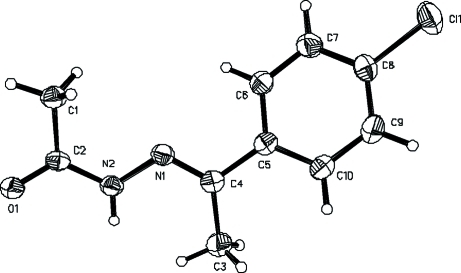
The molecular structure of the title compound with displacement ellipsoids drawn at the 30% probability level.

## supplementary crystallographic information

### Experimental

A mixture of 4-fluorobenzophenone (0.02 mol) and acethydrazide (0.02 mol) was stirred in refluxing ethanol(30 ml) for 2 h to afford the title compound (yield 82%).  Yellow bars of (I) were obtained by recrystallization from acetic ether at room temperature.

### Refinement

All H atoms were fixed geometrically and allowed to ride on their attached atoms, with C—H and N—H distances in the range 0.93–0.97Å and 0.86 Å, and with *U*_iso_(H) = 1.2*U*_eq_ of the parent atoms.

### Crystal data

**Table:**

C_10_H_11_ClN_2_O	*F*(000) = 440
*M**_r_* = 210.66	*D*_x_ = 1.333 Mg m^−^^3^
Monoclinic, *P*2_1_/*c*	Mo *K*α radiation, λ = 0.71073 Å
Hall symbol: -P 2ybc	Cell parameters from 2368 reflections
*a* = 15.944 (3) Å	θ = 3.1–27.5°
*b* = 5.0061 (10) Å	µ = 0.33 mm^−^^1^
*c* = 13.950 (3) Å	*T* = 293 K
β = 109.45 (3)°	Bar, yellow
*V* = 1049.9 (4) Å^3^	0.25 × 0.20 × 0.20 mm
*Z* = 4	

### Data collection

**Table:**

Bruker SMART CCD diffractometer	1840 reflections with *I* > 2σ(*I*)
Radiation source: fine-focus sealed tube	*R*_int_ = 0.028
graphite	θ_max_ = 27.5°, θ_min_ = 3.1°
phi and ω scans	*h* = −20→20
9225 measured reflections	*k* = −6→6
2368 independent reflections	*l* = −18→18

### Refinement

**Table:**

Refinement on *F*^2^	Primary atom site location: structure-invariant direct methods
Least-squares matrix: full	Secondary atom site location: difference Fourier map
*R*[*F*^2^ > 2σ(*F*^2^)] = 0.046	Hydrogen site location: inferred from neighbouring sites
*wR*(*F*^2^) = 0.164	H atoms treated by a mixture of independent and constrained refinement
*S* = 1.22	*w* = 1/[σ^2^(*F*_o_^2^) + (0.1*P*)^2^] where *P* = (*F*_o_^2^ + 2*F*_c_^2^)/3
2368 reflections	(Δ/σ)_max_ < 0.001
143 parameters	Δρ_max_ = 0.29 e Å^−^^3^
0 restraints	Δρ_min_ = −0.22 e Å^−^^3^

### Special details

**Table:**

Geometry. All e.s.d.'s (except the e.s.d. in the dihedral angle between two l.s. planes) are estimated using the full covariance matrix. The cell e.s.d.'s are taken into account individually in the estimation of e.s.d.'s in distances, angles and torsion angles; correlations between e.s.d.'s in cell parameters are only used when they are defined by crystal symmetry. An approximate (isotropic) treatment of cell e.s.d.'s is used for estimating e.s.d.'s involving l.s. planes.
Refinement. Refinement of *F*^2^ against ALL reflections. The weighted *R*-factor *wR* and goodness of fit *S* are based on *F*^2^, conventional *R*-factors *R* are based on *F*, with *F* set to zero for negative *F*^2^. The threshold expression of *F*^2^ > σ(*F*^2^) is used only for calculating *R*-factors(gt) *etc*. and is not relevant to the choice of reflections for refinement. *R*-factors based on *F*^2^ are statistically about twice as large as those based on *F*, and *R*- factors based on ALL data will be even larger.

### Fractional atomic coordinates and isotropic or equivalent isotropic displacement parameters (Å^2^)

**Table:**

	*x*	*y*	*z*	*U*_iso_*/*U*_eq_	
Cl1	0.05552 (4)	0.51605 (12)	0.20233 (5)	0.0784 (3)	
O1	0.58233 (7)	−0.3084 (2)	0.08042 (9)	0.0515 (3)	
N2	0.43948 (8)	−0.2513 (3)	0.06514 (10)	0.0437 (3)	
N1	0.37755 (9)	−0.1121 (3)	0.09450 (10)	0.0450 (3)	
C4	0.29443 (11)	−0.1619 (3)	0.04835 (12)	0.0449 (4)	
C5	0.23285 (11)	−0.0057 (3)	0.08658 (12)	0.0449 (4)	
C2	0.52624 (10)	−0.1844 (3)	0.10492 (11)	0.0409 (3)	
C8	0.12309 (12)	0.3099 (3)	0.15743 (15)	0.0544 (4)	
C7	0.20770 (13)	0.2495 (4)	0.22141 (14)	0.0598 (5)	
H7A	0.2280	0.3142	0.2877	0.072*	
C6	0.26121 (11)	0.0927 (4)	0.18559 (13)	0.0544 (4)	
H6A	0.3181	0.0507	0.2287	0.065*	
C10	0.14651 (12)	0.0543 (4)	0.02497 (14)	0.0572 (5)	
H10A	0.1251	−0.0132	−0.0408	0.069*	
C1	0.55118 (11)	0.0442 (3)	0.17832 (14)	0.0517 (4)	
H1B	0.6144	0.0700	0.2004	0.078*	
H1C	0.5332	0.0054	0.2360	0.078*	
H1D	0.5219	0.2036	0.1456	0.078*	
C9	0.09182 (12)	0.2137 (4)	0.06054 (15)	0.0620 (5)	
H9A	0.0344	0.2544	0.0186	0.074*	
C3	0.25881 (16)	−0.3524 (5)	−0.0385 (2)	0.0663 (6)	
H2A	0.4257 (13)	−0.391 (4)	0.0186 (17)	0.062 (5)*	
H3A	0.197 (2)	−0.372 (5)	−0.064 (2)	0.106 (9)*	
H3B	0.279 (3)	−0.503 (7)	−0.022 (3)	0.155 (16)*	
H3C	0.278 (2)	−0.321 (6)	−0.094 (2)	0.115 (10)*	

### Atomic displacement parameters (Å^2^)

**Table:**

	*U*^11^	*U*^22^	*U*^33^	*U*^12^	*U*^13^	*U*^23^
Cl1	0.0715 (4)	0.0887 (5)	0.0850 (5)	0.0221 (3)	0.0396 (3)	0.0014 (3)
O1	0.0511 (6)	0.0533 (6)	0.0489 (7)	0.0016 (5)	0.0151 (5)	−0.0108 (5)
N2	0.0479 (7)	0.0422 (7)	0.0401 (7)	0.0027 (6)	0.0134 (5)	−0.0058 (5)
N1	0.0475 (7)	0.0469 (7)	0.0399 (7)	0.0038 (6)	0.0137 (5)	−0.0018 (5)
C4	0.0495 (8)	0.0424 (8)	0.0417 (8)	−0.0018 (6)	0.0138 (6)	0.0016 (6)
C5	0.0462 (9)	0.0451 (8)	0.0414 (9)	−0.0024 (6)	0.0119 (7)	0.0029 (6)
C2	0.0510 (8)	0.0378 (7)	0.0325 (7)	0.0033 (6)	0.0122 (6)	0.0004 (5)
C8	0.0520 (9)	0.0554 (9)	0.0615 (11)	0.0043 (7)	0.0267 (8)	0.0037 (8)
C7	0.0601 (10)	0.0706 (11)	0.0469 (10)	0.0085 (9)	0.0154 (8)	−0.0030 (8)
C6	0.0501 (9)	0.0641 (10)	0.0442 (9)	0.0074 (8)	0.0092 (7)	−0.0016 (7)
C10	0.0466 (9)	0.0701 (11)	0.0496 (10)	−0.0019 (8)	0.0089 (7)	−0.0065 (8)
C1	0.0571 (10)	0.0488 (9)	0.0491 (10)	−0.0064 (7)	0.0175 (7)	−0.0115 (7)
C9	0.0429 (8)	0.0705 (11)	0.0663 (12)	0.0032 (8)	0.0097 (8)	0.0008 (9)
C3	0.0595 (12)	0.0661 (13)	0.0687 (14)	−0.0060 (10)	0.0154 (10)	−0.0225 (10)

### Geometric parameters (Å, °)

**Table:**

Cl1—C8	1.7512 (17)	C7—C6	1.370 (2)
O1—C2	1.2269 (18)	C7—H7A	0.9300
N2—C2	1.350 (2)	C6—H6A	0.9300
N2—N1	1.3773 (17)	C10—C9	1.390 (3)
N2—H2A	0.93 (2)	C10—H10A	0.9300
N1—C4	1.290 (2)	C1—H1B	0.9600
C4—C5	1.486 (2)	C1—H1C	0.9600
C4—C3	1.497 (2)	C1—H1D	0.9600
C5—C10	1.391 (2)	C9—H9A	0.9300
C5—C6	1.392 (2)	C3—H3A	0.93 (3)
C2—C1	1.498 (2)	C3—H3B	0.83 (4)
C8—C9	1.363 (3)	C3—H3C	0.94 (3)
C8—C7	1.381 (3)		
			
C2—N2—N1	119.36 (13)	C7—C6—H6A	119.1
C2—N2—H2A	116.5 (12)	C5—C6—H6A	119.1
N1—N2—H2A	124.2 (12)	C9—C10—C5	120.84 (17)
C4—N1—N2	118.22 (13)	C9—C10—H10A	119.6
N1—C4—C5	114.25 (13)	C5—C10—H10A	119.6
N1—C4—C3	125.23 (16)	C2—C1—H1B	109.5
C5—C4—C3	120.50 (15)	C2—C1—H1C	109.5
C10—C5—C6	117.66 (16)	H1B—C1—H1C	109.5
C10—C5—C4	121.86 (15)	C2—C1—H1D	109.5
C6—C5—C4	120.46 (14)	H1B—C1—H1D	109.5
O1—C2—N2	120.14 (14)	H1C—C1—H1D	109.5
O1—C2—C1	121.64 (14)	C8—C9—C10	119.41 (16)
N2—C2—C1	118.22 (14)	C8—C9—H9A	120.3
C9—C8—C7	121.30 (16)	C10—C9—H9A	120.3
C9—C8—Cl1	119.82 (14)	C4—C3—H3A	116.6 (17)
C7—C8—Cl1	118.88 (15)	C4—C3—H3B	110 (3)
C6—C7—C8	118.87 (17)	H3A—C3—H3B	106 (3)
C6—C7—H7A	120.6	C4—C3—H3C	115.3 (18)
C8—C7—H7A	120.6	H3A—C3—H3C	107 (2)
C7—C6—C5	121.89 (16)	H3B—C3—H3C	101 (3)

### Hydrogen-bond geometry (Å, °)

**Table:**

*D*—H···*A*	*D*—H	H···*A*	*D*···*A*	*D*—H···*A*
N2—H2A···O1^i^	0.93 (2)	2.02 (2)	2.9384 (18)	170.3 (18)

Symmetry codes: (i) −*x*+1, −*y*−1, −*z*.
